# Identification of a novel QTL and candidate gene associated with grain size using chromosome segment substitution lines in rice

**DOI:** 10.1038/s41598-020-80667-6

**Published:** 2021-01-08

**Authors:** Dianwen Wang, Wenqiang Sun, Zhiyang Yuan, Qiang Sun, Kai Fan, Chaopu Zhang, Sibin Yu

**Affiliations:** National Key Laboratory of Crop Genetic Improvement, College of Plant Science and Technology, Huazhong Agricultural University, Wuhan, 430070 China

**Keywords:** Genetics, Plant sciences

## Abstract

Rice is one of the staple crops in the world. Grain size is an important determinant of rice grain yield, but the genetic basis of the grain size remains unclear. Here, we report a set of chromosome segment substitution lines (CSSL) developed in the genetic background of the genome-sequenced *indica* cultivar Zhenshan 97. Genotyping of the CSSLs by single nucleotide polymorphism array shows that most carry only one or two segments introduced from the genome-sequenced *japonica* cultivar Nipponbare. Using this population and the high-density markers, a total of 43 quantitative trait loci were identified for seven panicle- and grain-related traits. Among these loci, the novel locus *qGL11* for grain length and thousand-grain weight was validated in a CSSL-derived segregating population and finely mapped to a 25-kb region that contains an IAA-amido synthetase gene *OsGH3.13*, This gene exhibited a significant expression difference in the young panicle between the near-isogenic lines that carry the contrasting Zhenshan 97 and Nipponbare alleles at *qGL11*. Expression and sequence analyses suggest that this gene is the most likely candidate for *qGL11*. Furthermore, several *OsGH3.13* mutants induced by a CRISPR/Cas9 approach in either *japonica* or *indica* exhibit an increased grain length and thousand-grain weight, thus enhancing the final grain yield per plant. These findings provide insights into the genetic basis of grain size for the improvement of yield potential in rice breeding programs.

## Introduction

Rice (*Oryza sativa* L.) is the staple food for more than half of the world’s population. Rice yield is highly correlated with number of panicles per plant, number of seeds per panicle, and thousand-grain weight (TGW). Grain size (or grain weight) is mainly determined by its three dimensions: the grain length (GL), the grain width (GW), and the grain thickness. Due to its importance in yield improvement, panicle and grain traits have been extensively studied by genetic and molecular analyses. To date, a large number of quantitative trait loci (QTL) for these traits have been reported in rice (http://www.gramene.org/). Numerous genes associated with grain size have been identified, involving in multiple regulation pathways, such as the ubiquitin–proteasome pathway, G-protein signaling, mitogen-activated protein kinase signaling, the HAIKU (IKU) pathway, phytohormone perception and homeostasis^1–4^. As an essential phytohormone, Auxin (IAA) regulates a very broad range of plant growth and developmental processes. Many auxin signaling and transport genes have been reported to control grain size. For example, *qTGW3*, a major QTL for grain weight, encodes a SHAGGY-like kinase 41 (*OsSK41*)/*OsGSK5*, which interacts with auxin response factor 4 (*OsARF4*) to negatively regulate grain size and grain weight by affecting cell expansion^5^. *OsSK41* and *OsARF4* regulate the expression of some auxin-response genes. *BIG GRAIN1* (*BG1*) encodes a positive regulator of auxin response and transport, which markedly increased the indole-3-acetic acid (IAA) level in the panicle. The activation of *BG1* results in the production of large grains^6^. In addition, *TGW6*, a major QTL for thousand-grain weight, encodes an IAA-glucose hydrolase that produces free IAA^7^. Loss of function of *TGW6* enhances grain weight and increases yield in rice. Although some genes in the auxin pathway have been reported as influencing grain size, the current understanding of the role of auxin (IAA) inactivation in the regulation of grain size is limited.

The identification of QTLs is a first step toward dissecting the genetic and molecular bases of grain size. Several studies have proven that chromosome segment substitution lines (CSSL) as a permanent genetic resource serve as a powerful platform for the identification, fine mapping and cloning of QTLs underlying complex traits^8–11^. Each CSSLs contains one or a few substitution segments of a particular donor genome within the common genetic background, providing a great advantage in identifying minor-effect QTLs, mainly due to minimization of the genetic interaction or background noise. CSSL could also facilitate the rapid development of near-isogenic lines (NIL) and secondary segregating populations for further fine mapping and cloning of the QTLs of interest^12–15^. This research process can be accelerated by high-throughput single nucleotide polymorphism (SNP) approaches. Therefore, a large number of CSSL populations have been developed with great effort in rice and other crops. However, most of the populations are composed of CSSLs each with multiple substituted segments^16^, although many populations were constructed with high-density mapping^17,18^.

In this study, we developed a set of genotype-defined CSSLs derived from a cross of two genome-sequenced varieties, Nipponbare (NIP), as a *japonica* cultivar^19^, and Zhenshan 97 (ZS97), as an *indica* cultivar^20^. Using the developed CSSLs with high-density SNP markers, we performed QTL analysis for seven panicle and grain-related traits and identified a number of QTLs with minor effects on the assayed traits. Furthermore, a novel QTL (*qGL11*) for grain length was validated and finely mapped to a small region containing *OsGH3.13*, an IAA-amido synthetase, specifically catalyzing the conversion of active IAA to its inactive form via the conjugation of IAA with amino acids^21,22^. The CRISPR-generated *OsGH3.13* mutants exhibited increased grain length and grain weight, indicating that the regulation of grain size involves the IAA metabolic pathway*.*

## Results

### Development of the CSSL population with SNP-defined genotypes

Previously, a NIP/ZS97 CSSL population consisting of 143 lines (named original population) was constructed using an marker-assisted selection (MAS) backcross scheme^23,24^. However, most of the CSSLs contained two or more NIP segments when genotyped using an Infinium RICE6K array^24^. Therefore, we further used the MAS backcross scheme to improve the uniformity of the backgrounds of the CSSLs. Thirty-four lines, each with fewer than 4 substitution segments, were preferentially selected to backcross with ZS97 and self-cross to generate BC_1_F_2_. In each BC_1_F_2_ population, approximately 100 individuals were genotyped with simple sequence repeats (SSR) and insertion-deletion (Indel) markers targeting a particular substitution segment and background segments. Then, a total of 87 heterozygous and 88 homozygous individuals that contained only a single substitution segment of corresponding chromosomal regions were obtained. For the 87 plants heterozygous at a particular substitution segment, an F_2_ progeny of 20 individuals was planted to select a desirable homozygous genotype at the target region (Supplementary Fig. S1). Finally, the updated CSSL population comprising 175 new lines and 46 original lines was developed, in which all substitution segments together almost covered the entire *japonica* genome. To define the genotypes of the introduced segments more precisely, this newly developed CSSL population (named as DNZ) was subjected to genotyping with the Rice 8 K SNP array.

The SNP genotyping of the DNZ population revealed 3,610 polymorphic SNPs between NIP and ZS97 evenly distributed across the 12 rice chromosomes, with a median density of 50.3 kb per adjacent SNP (Supplementary Table S1). The population comprises 142 (64%) lines with only a single substitution segment, 50 (23%) lines with two substitution segments, and 29 lines with three or more introduced segments (Supplementary Fig. S2), which in total contain 408 substitution segments with an average length of 2.3 Mb, covering 96% of the NIP genome (Supplementary Table S2). Based on the recombination breakpoints caused by the 408 substituted segments, a bin map contains 418 bins ranging from 1.8 kb to 8.2 Mb with a median size of 473 kb across the whole genome was constructed for the DNZ population (Supplementary Table S2).

### Phenotypic variation of panicle and grain traits

**Figure 1:**
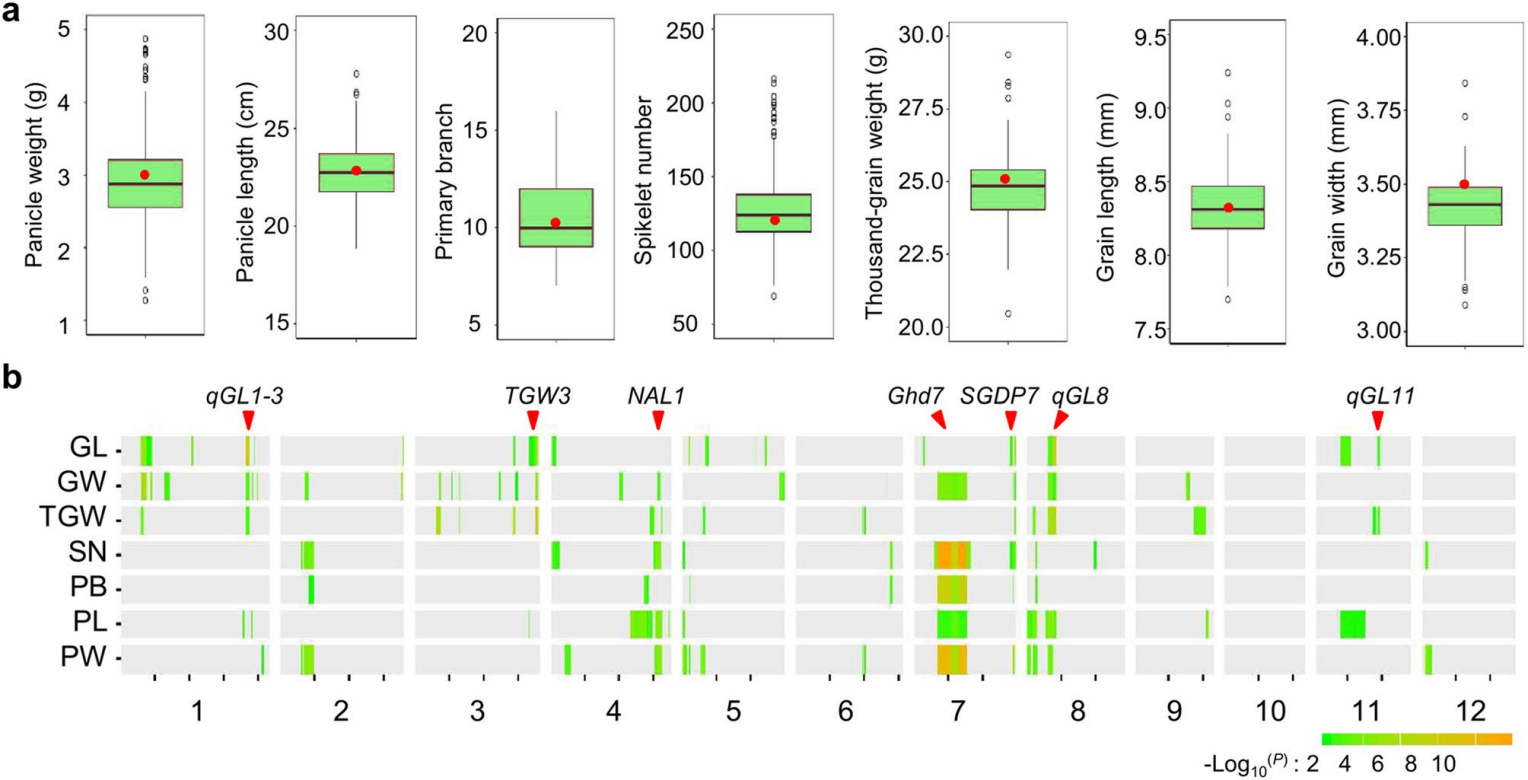
Phenotypic and QTL analysis of the DNZ CSSL population. (**a**) Variation of the phenotypic traits in the DNZ CSSL population. In the box plots, the horizontal line within the box indicates the median value; the bars of the box indicate the limits as 1.5 times the interquartile range from the box; the dots outside the bars indicate the most extreme data points or possible outliers. The red dot indicates the phenotype value of ZS97. (**b**) Overviews of QTL results for seven traits in the DNZ CSSL population. Rectangle color density indicates the magnitude of -log_10_ (*P*) values in the ridge regression significance test. The horizontal position of the rectangle indicates the relative physical position of QTL support interval on the chromosome. The triangle indicates approximately physical location of a known gene for grain size. GL, GW, TGW, SN, PB, PL and PW represent grain length, grain width, thousand-grain weight, spikelet number, primary branch, panicle length and panicle weight, respectively. The sub-figures (**a**, **b**) were prepared using R software (version 3.5.2) (http://www.R-project.org/).

Two parents (NIP and ZS97) exhibited significant differences in panicle and grain traits, with lower values in NIP than ZS97 for all seven assayed traits, except for primary branch (Supplementary Table S3). The frequency distribution of these traits all showed a large continuous variation in the DNZ population (Supplementary Fig. S3), indicating that the panicle and grain-related traits were in quantitative inheritance controlled by polygenes or QTLs. Most of the lines were similar to the parent ZS97 in all assayed traits (Fig. 1a; Supplementary Table S3); however, several lines exhibited phenotypic values surpassing ZS97 at various degree in both directions (Fig. 1a), which suggests that some QTLs or genes located in the corresponding substitution segments had either negative or positive effects on the panicle and grain traits.

### Detection of QTLs for the panicle and grain traits

The ridge regression analyses for the QTL detection were performed in the DNZ population and the original population with the bin genotypes. In total, 43 bins containing 89 QTLs were identified for seven panicle and grain traits (Supplementary Table S4), and 37 bins containing 82 loci detected for these traits in the original population. The same or overlapping bin carrying several QTLs for multiple traits was considered as only one QTL. Hence, 43 bin QTLs were identified in the DNZ population. Most (24/43) of these loci were repeatedly detected in the two populations (Supplementary Table S5), suggesting their stable effects in different environments. Therefore, the QTLs identified in the DNZ population are present below in details.

Among the loci in the DNZ population, 18 were located in bins with sizes of less than 500 kb, and 7 loci were mapped in much smaller bins (less than 200 kb) (Supplementary Table S4). The QTLs detected in small bins were significant for further fine mapping and candidate gene pinpointing. The phenotypic variation explained (PVE) by each QTL ranged from 0.7 to 16.9%, in which only three bin QTLs had major-effect (PVE ≥ 10%), while other bins containing 70 loci exhibited minor effects (PVE < 5%) (Supplementary Table S4). These results confirm that the panicle and grain traits are complex quantitative traits controlled by multiple genes. The majority of the QTLs exhibited a positive effect, with the NIP alleles increasing panicle and grain size (Supplementary Table S4).

Regarding grain size, 32 bins containing 49 QTLs were identified distributed on all chromosomes except chromosome 12 in the DZN population (Supplementary Table S4). Several QTLs were identified in common for grain length, grain width and thousand-grain weight, which is consistent with the significant correlation between these three traits (Supplementary Fig. S3). For example, three QTLs, *qGL1-3*, *qGW1-3* and *qTGW1-2*, were colocalized in Bin029 on chromosome 1. The loci *qGL3-2, qGW3-5* and *qTGW3-4* mapped in Bin147 on chromosome 3 were colocalized in the same region surrounding *TGW3*^5,25,26^ (Fig. 1b). Three loci, *qGL8* and *qTGW8-2* with the largest effect on grain length or thousand-grain weight, along with *qGW8*, were mapped in Bin329 (approximately 354 kb) on chromosome 8, which may be the same QTL for all three grain traits (Fig. 1b; Supplementary Table S4). The loci *qGL11* for grain length and *qTGW11* for thousand-grain weight were mapped in Bin390 on chromosome 11*.*

Similarly, there were 21 bins harboring 40 QTLs for panicle architecture (Supplementary Table S4). Four loci (*qPL4-2, qSN4-2*, *qPW4-2*, and *qPB4-2*) were detected for panicle architecture in the same region surrounding *NAL1*, which was previously reported as affecting panicle length, spikelet number and panicle weight^27^. Two QTLs on chromosomes 5 and 7 were identified in common for four panicle-related traits (Supplementary Table S4). Four loci (*qPB7-1*, *qPL7*, *qSN7-1* and *qPW7-1*), detected for primary branch, panicle length, spikelet number and panicle weight, respectively, were colocalized in the same region (Bin295) encompassing *Ghd7*, a cloned gene regulating multiple yield-related traits^28^. Three QTLs (*qPW7-2*, *qSN7-2* and *qPB7-2*) were co-localized in Bin309 (appropriately 110 kb) containing *SGDP7*, a gene for small grain and dense panicle^29^. The colocalization of these QTLs is a genetic refection of the significant correlation between the panicle-related traits (Supplementary Fig. S3).

### Fine mapping of *qGL11* for grain length

**Figure 2:**
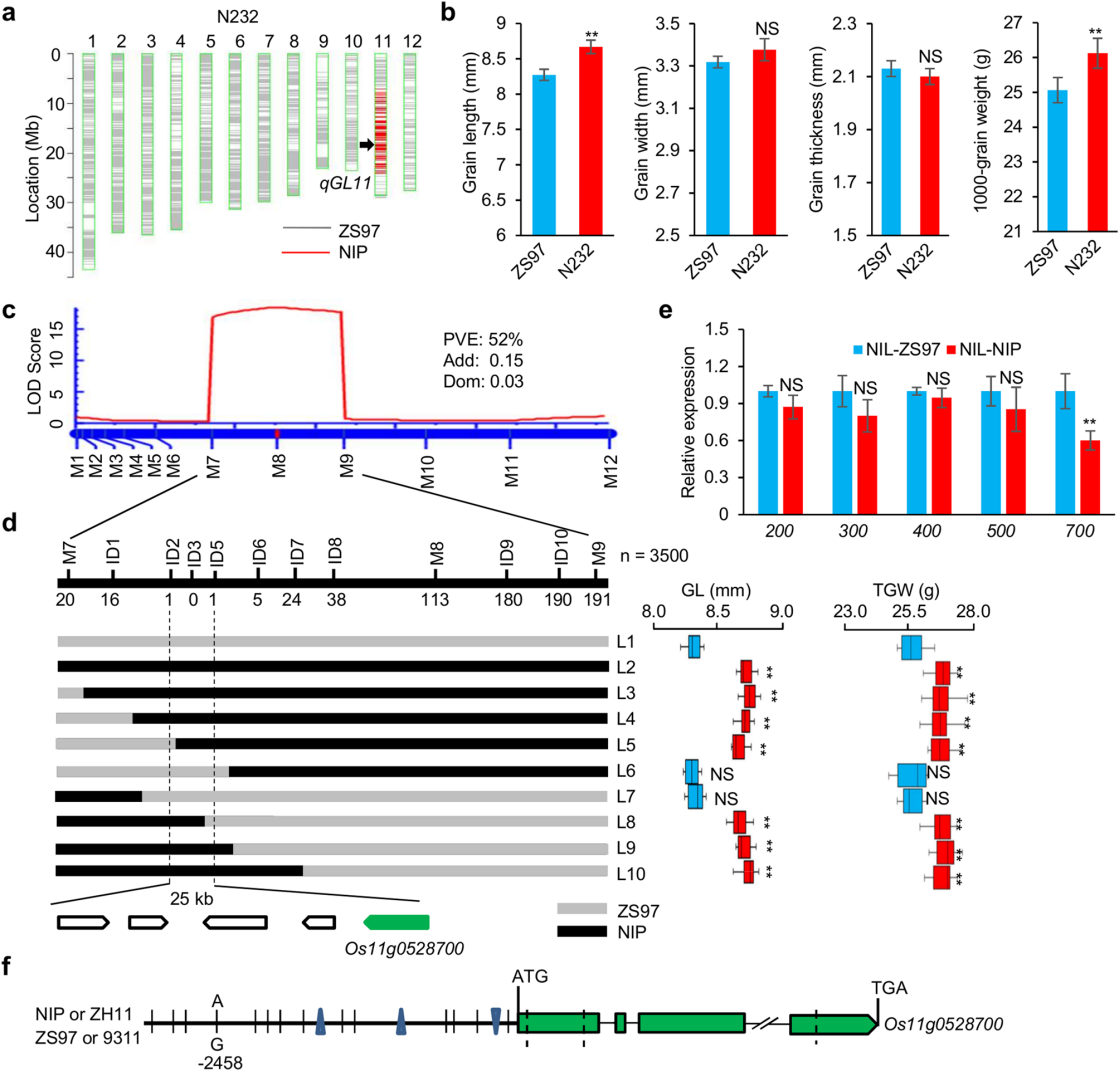
Fine mapping of *qGL11*. (**a**) Graphic genotype of N232 showing a single substitution segment encompassing *qGL11*. (**b**) Comparison of the grain size in ZS97 and N232. Differences between ZS97 and N232 in grain length, grain width, grain thickness and thousand-grain weight. Data are given as the mean and SE (*n* = 6 individuals). (**c**) The QTL was detected in a CSSL-derived F_2_ population. PVE, Add and Dom respectively represent phenotypic variation explained, additive effect and dominant effect. (**d**) Fine mapping of *qGL11* in a 25-kb region using a large segregating population (*n* = 3500). Five predicted genes are indicated, including *Os11g0528700* (700) which was selected as the candidate gene. L3-L10 represent recombinant plants. Phenotype data for GL (grain length) and TGW (thousand-grain weight) are provided as the mean and SE (*n* = 10). (**e**) Quantitative RT-PCR analysis of gene expression in the 8-cm panicle. Transcription levels relative to NIL-ZS97, which was set to 1, are presented as the mean and SE of triplicates. *LOC_Os03g13170* (*Ubiquitin*) is the control gene. (**f**) Sequence comparison of *Os11g0528700* among four cultivars. The triangle indicates a nucleotide deletion. **, * and NS indicate significant differences at *P* < 0.01, *P* < 0.05, and no significance, respectively, by Student’s *t*-test.

To verify a newly identified locus (*qGL11* or *qTGW11*), a CSSL (named N232) carrying a single substitution segment covering *qGL11* was crossed with ZS97 to generate a CSSL-derived F_2_ population. N232 had a longer grain length and larger grain weight than ZS97 (Fig. 2a,b). First, a small F_2_ population comprising 107 individuals was genotyped using 12 polymorphic markers in this segment (Supplementary Table S6). The QTL analysis in this population revealed that *qGL11* was located in the interval between markers M7 and M9, explaining 52% of the phenotypic variance for grain length. The additive effect of the NIP alleles was 0.15, and the dominant effect was 0.03 (Fig. 2c). Then, a large CSSL-derived segregating population comprising 3500 individuals was used to select recombinants between M7 and M9 by using ten additional Indel markers (Supplementary Table S6). Based on the progeny phenotyping of grain size for the informative recombinants, *qGL11* was delimited to a 25-kb region flanked by markers ID2 and ID5 (Fig. 2d). This region contains five predicted genes according to the RAP database^30^ (https://rapdb.dna.affrc.go.jp/). Among the five annotated genes, only *Os11g0528700* (*OsGH3.13*), encoding an indole-3-acetic acid (IAA)-amido synthetase, exhibited a significant expression difference in the young panicle between a pair of NILs (NIL-NIP and NIL-ZS97), which carry a single NIP segment (less than 2 Mb) covering the contrasting NIP and ZS97 alleles at *qGL11* within the ZS97 background (Fig. 2e). Sequence comparison revealed that *OsGH3.13* had substantial variation among rice varieties. In its promoter region (3-kb upstream of the predicted start codon), 14 SNPs and three Indels were found between ZS97 and NIP. In particular, the A/G variant at the -2458 site upstream of the start codon may cause auxin-response element (GATACA) defects in *OsGH3.13*^NIP^ (Fig. 2f). In the coding region, there were three nonsynonymous SNPs resulting in amino acid changes (Supplementary Table S7). Thus, *OsGH3.13* is a possible candidate gene for *qGL11*.

### Validation of the *qGL11* effect in NILs

**Figure 3:**
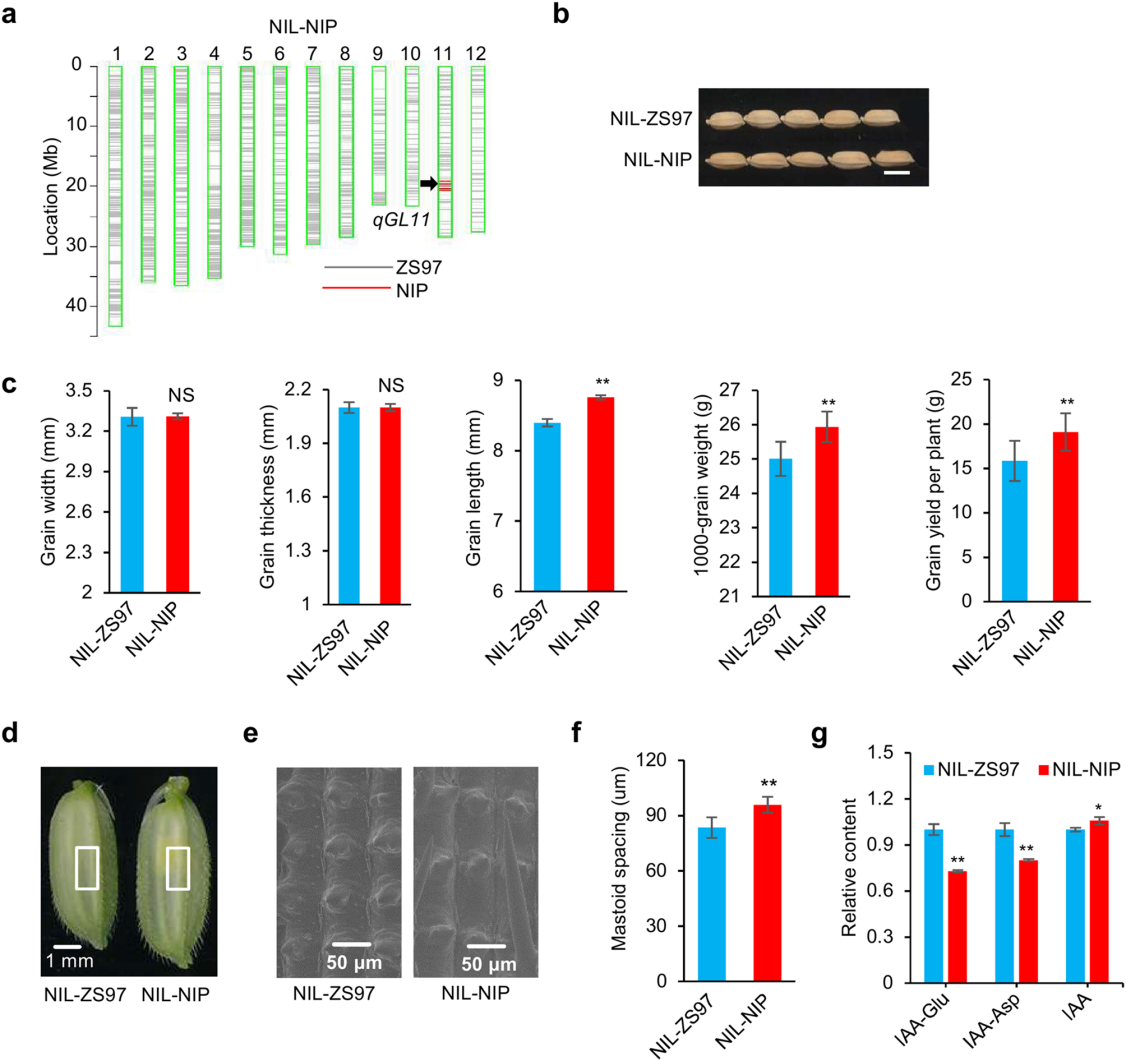
Differences between NIL-ZS97 and NIL-NIP with the contrasting alleles at *qGL11.* (**a**) Graphic genotype representation of NIL-NIP. (**b**) Differences between NIL-ZS97 and NIL-NIP in seed morphology. Scale bar: 5 mm. (**c**) Comparison of the grain size and grain yield in NIL-ZS97 and NIL-NIP. Data are given as the mean and SE (*n* = 10). (**d**–**f**) Scanning electron microscopy analysis of the lemma. Differences between NIL-ZS97 and NIL-NIP in spikelet morphology (**d**), cell morphology (**e**), longitudinal cell length (**f**) in the lemmas of spikelet hulls. Values provided as the mean and SE (*n* = 10). (**g**) IAA and IAA-amino acid conjugate species in NIL-ZS97 and NIL-NIP. Content relative to NIL-ZS97, which was set to 1, is presented as the mean and SE of triplicates. **, * indicate a significant difference at *P < 0.01* and *P* < 0.05, respectively, by Student’s *t*-test*.* NS, no significance.

To confirm the *qGL11* effect, NIL-NIP and NIL-ZS97 were evaluated in grain size and other yield-related traits (Fig. 3a). Phenotypic comparison of NILs showed that the average grain length of NIL-NIP was 4.3% longer than that of NIL-ZS97, and the thousand-grain weight was 3.7% greater than that of NIL-ZS97, while grain width and grain thickness was not significantly different between NILs (Fig. 3b,c). Notably, the grain yield of NIL-NIP was increased by approximately 20% compared to that of NIL-ZS97 (Fig. 3b). The effective panicle number was slightly increased, but not significantly, between NILs (Supplementary Fig. S4). Other agronomic traits, such as heading date, plant height, panicle length, primary branch, spikelet number, and seed setting ratio, also showed no significant difference between the two NILs (Supplementary Fig. S4). To investigate whether cell expansion was involved in NILs, scanning electron microscopy analysis was conducted and revealed that NIL-NIP had an average mastoid spacing of 95.9 ± 4.4 µm, significantly larger than those of NIL-ZS97 (average 83.5 ± 5.6 µm). The NIL-NIP epidermal cells were approximately 19.2% longer than their counterparts (Fig. 3d–f), suggesting that the longer cells of NIL-NIP largely accounted for the differences in grain size. It has been reported that *OsGH3.13* is an IAA-amido synthetase, specifically catalyzing the conversion of active IAA into its inactive form via the conjugation of IAA with amino acids^21,22^. Liquid chromatography-mass spectrometry analyses were thus performed to measure the IAA and its conjugates in NILs. The results revealed higher free IAA concentration in NIL-NIP than in NIL-ZS97. Consistent with this observation, the relative contents of two IAA conjugates IAA-Glu and IAA-Asp were lower in NIL-NIP than in NIL-ZS97 (Fig. 3g).

**Figure 4:**
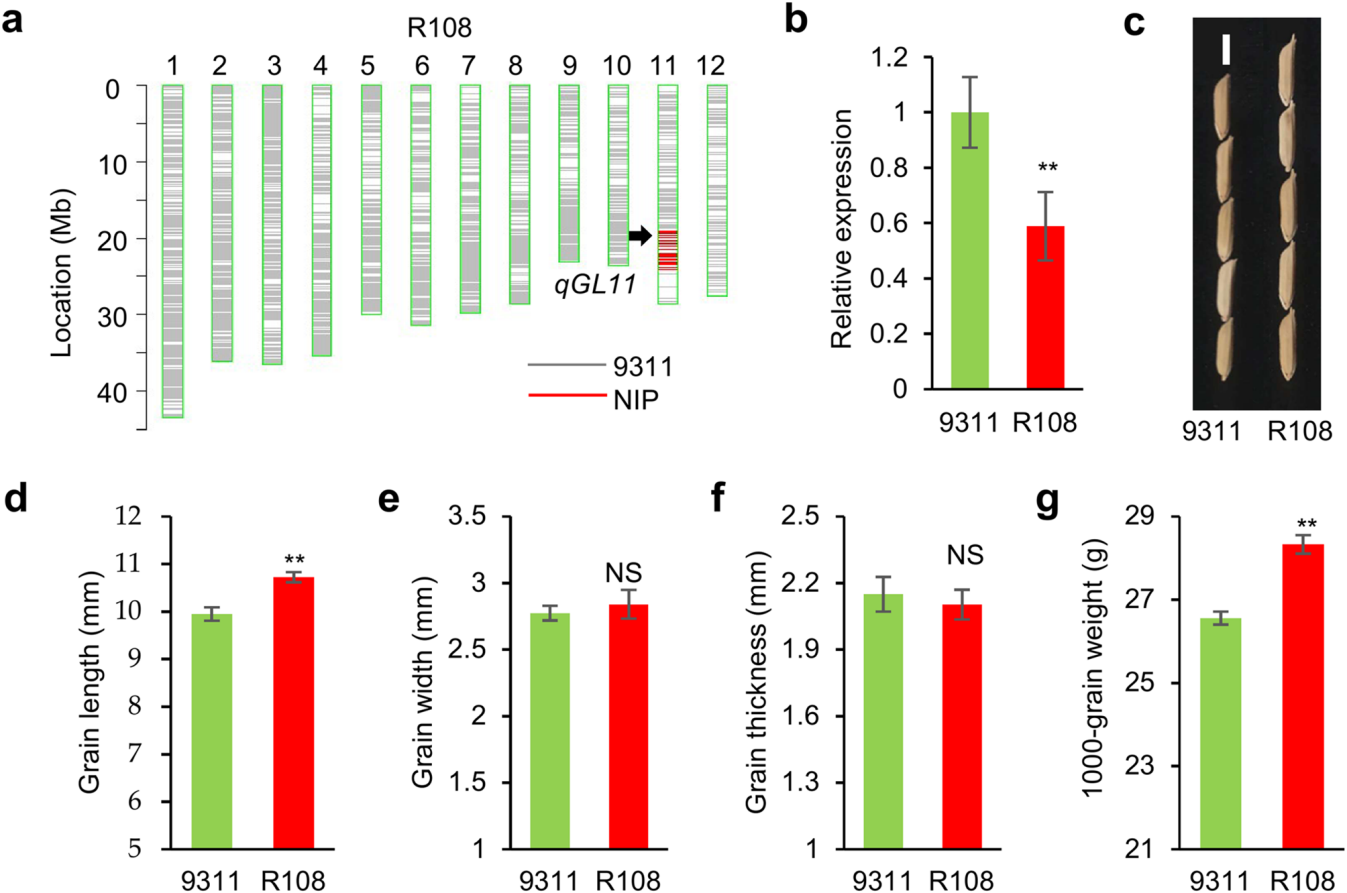
Comparative analysis of NILs containing *qGL11* in the background of 9311. (**a**) Graphic representation of the genotype of R108 that carries a single substituted segment encompassing *qGL11*. (**b**) Relative expression of *OsGH3.13* of 9311 and R108 in the 8-cm panicle. Transcription levels relative to 9311, which was set to 1, are presented as the mean and SE (*n* = 3). *Ubiquitin* is the control gene. (**c**–**g**) Differences between 9311 and R108 in seed morphology (**c**), scale = 5 mm, grain length (**d**), grain width (**e**), grain thickness (**f**) and thousand-grain weight (**g**). (**d**–**g**) Data are given as the mean and SE (*n* = 6). ** and NS indicate statistically significant differences by Student’s *t*-test at *P* < 0.01 and no significance, respectively.

Sequence comparison of *OsGH3.13* among four varieties, NIP, ZS97, 9311 and ZH11, revealed that ZH11 and NIP had the same sequence, while 9311 and ZS97 shared the same sequence (Fig. 2f). Therefore, the other NIL (R108) that harbors a single NIP segment (less than 5 Mb) encompassing *qGL11* in the 9311 background (Fig. 4a) was also evaluated. Similarly, *OsGH3.13* exhibited a lower expression in R108 than in 9311 (Fig. 4b). Consistent with the grain size difference between NIL-NIP and NIL-ZS97, R108 revealed longer grain length and larger thousand-grain weight than 9311, with no significant difference in grain width and grain thickness (Fig. 4c–g). Taken together, these results indicate that *qGL11*^*NIP*^ is a positive factor, increasing grain length and grain weight in both the ZS97 and 9311 genetic backgrounds.

### *OsGH3.13* mutants increased grain size

**Figure 5:**
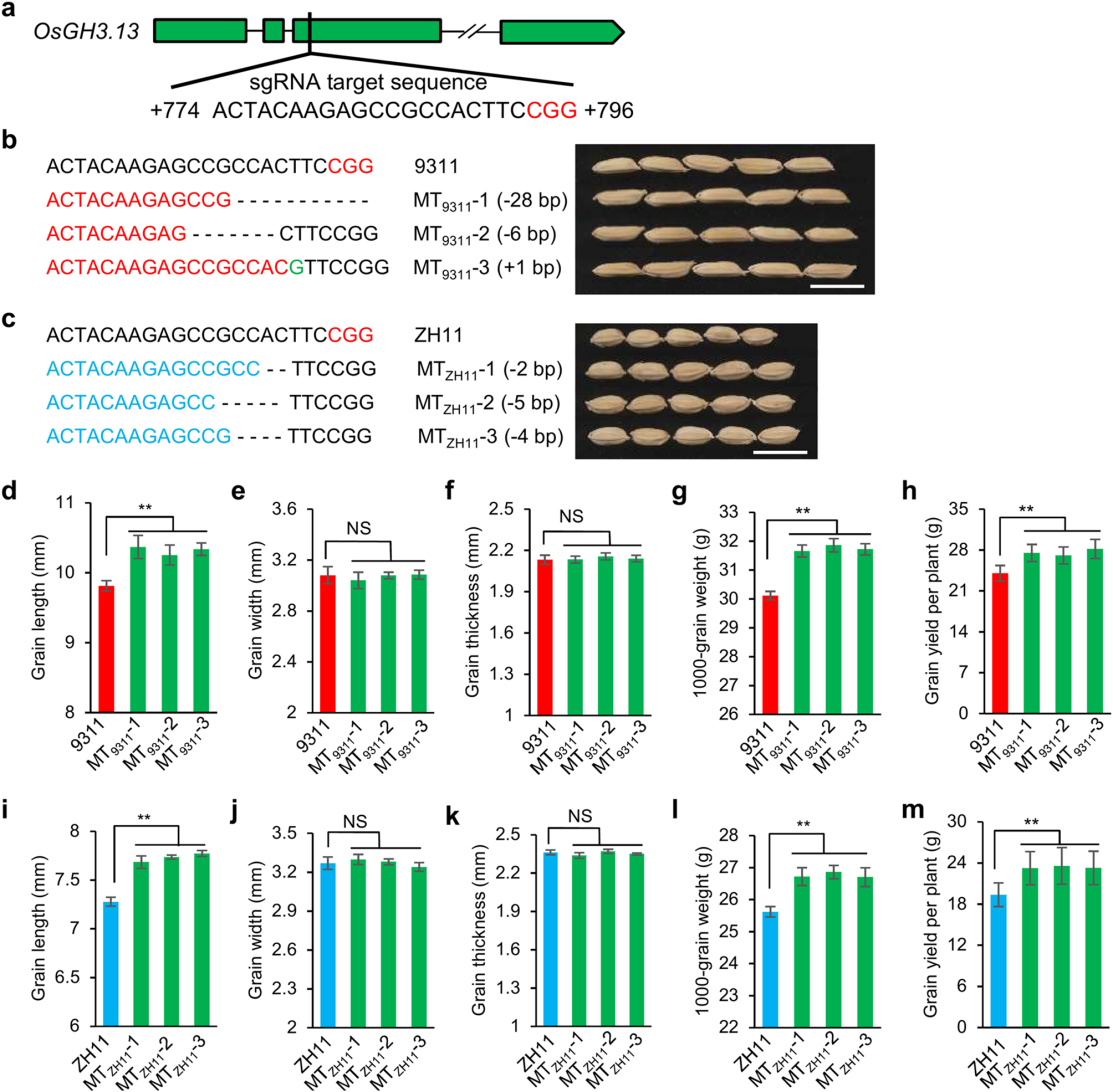
CRISPR/Cas9-induced mutants of the gene and their phenotypes. (**a**) Schematic gene model of *OsGH3.13* showing the sgRNA target site and sequence. The protospacer-adjacent motif (CGG) is shown in red font. (**b**, **c**) Homozygous mutations of *OsGH3.13* in 9311 (**b**), and ZH11 (**c**). Scale bars: 10 mm. (**d**–**m**) Differences between the mutants and corresponding wild type in grain size and grain yield per plant. Data are given as the mean and SE (*n* = 6). Asterisks (**) indicate significant difference from the wild type by Student’s *t*-test at *P* < 0.01. NS, no significance.

To determine whether *OsGH3.13* affects grain size, several mutants of *OsGH3.13* were generated separately in 9311 and ZH11 by using the CRISPR/Cas9 strategy (Fig. 5a–c). A total of 22 mutants were first verified as positive transgenic plants by PCR using specific *Cas9* primers, and sequence analysis identified the nucleotide mutations produced in the target exon of the gene in the positive plants. Finally, six homozygous independent mutants of *OsGH3.13* with various variants were obtained and characterized in the corresponding genetic background of ZH11 or 9311 (Fig. 5b,c). Three independent mutants in 9311 (MT_9311_-1, MT_9311_-2, MT_9311_-3), which carried a 28-bp deletion, 6-bp deletion and 1-bp insert in the target region, respectively. The 28-bp deletion and 1-bp insert resulted in frameshifts causing a premature termination of transcription of the gene, while the 6-bp deletion might cause an encoded incomplete peptide. As expected, these mutants exhibited increased grain length and thousand-grain weight compared to 9311 (Fig. 5d–h). Similarly, three independent mutations in ZH11 (MT_ZH11_-1, MT_ZH11_-2, MT_ZH11_-3) exhibited longer grain length and larger grain weight compared to ZH11 (Fig. 5i–m). All six mutants significantly increased average grain yield per plant by 14–20% compared to the counterpart 9311 or ZH11 while not influencing the other agronomic traits, such as plant height, effective panicle number, primary branch, panicle length, spikelet number, and seed setting ratio (Supplementary Fig. S5). These results indicate that *OsGH3.13* could functionally regulate grain length, thousand-grain weight, and final grain yield in rice.

## Discussion

It has been reported that CSSLs as an advanced backcross population can effectively reduce the interaction between QTLs and minimize the genetic background interference, thus improving the power to detect, fine-map and clone QTLs^31–36^. However, most CSSLs carry multiple and long substituted chromosomal segments with a low marker density, which could decrease the detection power, especially for those minor QTLs that are much more easily masked by genetic interactions. In this study, we developed NIP/ZS97 CSSLs with high-density SNPs and identified the role of *OsGH3.13* in affecting grain size*.* This updated set of CSSLs includes 192 (87%) lines containing only one or two substitution segments (Supplementary Fig. S2), facilitating the identification of the QTLs of complex traits. In this study, a large number of minor QTLs were detected for panicle and grain traits. Many QTLs are mapped in the same or overlapping regions of known genes or QTLs previously reported in rice (Supplementary Table S4). Moreover, a minor QTL, *qGL11* for grain length, was identified and finely mapped using the CSSL population and a CSSL-derived secondary segregating population (Fig. 2). These data indicate that CSSLs with high-density SNPs can provide an efficient tool for the identification of minor-effect QTLs associated with yield-related traits in rice, which will be of great value for rice breeding.

One of the notable findings of this study is that *OsGH3.13* affects grain length and thousand-grain weight in rice through regulating the IAA level. IAA is deactivated through either conjugation to amino acids or chemical oxidation, which has critical roles in maintaining local auxin homeostasis and normal plant development^37^. *OsGH3.13* is an IAA-amido synthetase, which catalyzes the conjugation of IAA to amino acids. Previously, *TLD1* was reported as *OsGH3.13*, downregulating IAA content, resulting in IAA deficiency and dramatic changes in plant architecture and enhancing rice drought tolerance^22^. A gain-of-function rice mutant (*tld1-D*) increased number of tillers, enlarged leaf angles, and dwarfism in rice. However, the loss-of-function mutant *tld1/OsGH3.13* did not show visible differences in plant growth as well as grain size from wild-type plants. The *OsGH3.13* homologous genes *OsGH3.8* and *OsGH3.1* were described as participating in disease resistance to rice pathogens^38,39^. Overexpression of *OsGH3.8* suppressed cell wall relaxation and inhibited plant growth^38^. In this study, we used a CSSL-derived segregating population to narrow down *qGL11* to a 25-kb region that contains the gene *OsGH3.13* (Fig. 2). We find that *OsGH3.13*^*NIP*^ has IAA-conjugating activity, leading to an increase of IAA and a decrease of IAA-Asp and IAA-Glu in NIL-NIP relative to NIL-ZS97 (Fig. 3). Sequence analysis shows several allelic variations, namely, *OsGH3.13*^*NIP*^*, OsGH3.13*^*ZS*^*,* and *OsGH3.13*^*9311*^. In particular, the A/G variant at the site -2458 upstream of the gene start codon results in a defective motif of GATACA^40^, which may cause a significant difference in expression level. In agreement with the expression variation, NIL-NIP (or R108), in which *OsGH3.13* was expressed at a low level, exhibits an increased grain length and grain weight compared to NIL-ZS97 (or 9311), in which the gene is expressed at a high level (Figs. 2e, 4b). Moreover, the CRISPR-induced *OsGH3.13* mutants in two genetic backgrounds (9311 and ZH11) confirmed that the function of *OsGH3.13* increased grain length and grain weight, and even the final grain yield per plant (Fig. 5). As *OsGH3.13* underlying *qGL11* could not affect grain width and grain thickness, we assume that the QTL/gene affects grain weight mainly by regulating grain length, although the regulation mechanism is required further investigation. In addition, *OsGH3.13*, like *TGW6* (an IAA-glucose hydrolase gene), is involved in auxin homeostasis influencing grain size. However, *OsGH3.13* influences grain length and weight with an effect on husk size (Fig. 3). It is different from *TGW6*, which regulates grain weight with no effect on husk size^7^. Our data establish a connection between IAA-amido synthetase and grain size and present the feasibility of modulating IAA homeostasis to influence grain size. Taken together, these results indicate that *OsGH3.13* corresponds to *qGL11* for grain size, with the *OsGH3.13*^*NIP*^ alleles exhibiting a positive effect on grain length and grain weight. These naturally favorable alleles can increase grain yield without affecting other agronomic traits and have great potential in rice breeding for yield improvement in rice.

## Materials and methods

### Plant materials

Previously, a CSSL population consisting of 143 lines was generated from a cross of the genome-sequenced cultivars ZS97 and NIP^23,24^. As some lines of the original population carried multiple introgressed NIP segments, it was necessary to develop the lines each with only single NIP segment of a particular chromosomal region in the ZS97 background. Therefore, a new CSSL population (named DNZ) comprising 221 lines was developed by a backcrossing scheme with MAS (Supplementary Fig. S1) and used for further study.

A CSSL-derived F_2_ population segregating at the target region was generated for fine mapping. In the process of fine mapping, the pairwise NILs (e.g. NIL-NIP and NIL-ZS97) that carry the contrasting NIP and ZS97 alleles at the QTL of interest within the common background of ZS97 were generated from the heterozygous recombinant line that contains the smallest introgression segment covering the QTL. The line R108 was also selected from the CSSL population derived from a cross of NIP as the donor and the cultivar 9311 as the recurrent parent for the QTL confirmation^41^.

### SNP genotyping and bin mapping of QTL

The DNZ population was genotyped using the rice 8 K array, which contains 7,519 SNP, by the China Golden Marker Co. (Beijing, China). Based on the SNP genotyping, a bin map was constructed for the CSSL population. The QTL analyses with the bins as markers were performed using linear ridge regression in R software with the package “ridge” (http://www.R-project.org/)^42^, as described previously^24^. A significance level of *P* < 0.01 was set as the threshold for the QTL declaration. The phenotypic variance explained by each QTL was decomposed by using ‘lmg’ from the ‘relaimpo’ package^42^. QTL nomenclature followed the principles as suggested^43^. The QTL IciMapping V4.2 (http://www.isbreeding.net/) was applied for the QTL validation in the CSSL-derived segregating population.

### DNA extraction and marker analysis

Genomic DNA from fresh young leaves of each line was extracted using the CTAB method^44^. Polymorphic SSR and Indel markers were used for MAS, The SSRs were selected from Gramene Database^45^, and Indel markers were developed according to the sequence variation between NIP and ZS97 (http://ricevarmap.ncpgr.cn)^46^. The primers were designed according to the Nipponbare reference genome by Primer3 (http://redb.ncpgr.cn/modules/redbtools/primer3.php). SNP markers in a given region were detected by sequencing. The sequences were analyzed using Sequencer 5.0 (Gene Codes Corporation). All primers were synthesized at Sangon Biotech (Shanghai) and are listed in Supplementary Table S6.

### Quantitative real-time PCR analysis

Total RNAs were isolated with the TRIzol kit (Invitrogen, CA) according to the manufacturer’s instructions. The RNA was treated with DNase I (Invitrogen), and approximately 3 μg of total RNA was used to synthesize first-strand cDNA using oligo (dT)_18_ as primer (Promega, Shanghai). Quantitative real-time (qRT) PCR was performed using gene-specific primers (Supplementary Table S6) and the FastStart Universal SYBR Green Master (Roche) on a real-time PCR ViiA7 system (Applied Biosystems). The rice *Ubiquitin* gene was used as the internal control. The relative quantification method was used to evaluate gene expression level^47^. At least three biological replicates were performed for each experiment.

### Trait measurement

All plant materials except the original CSSLs were planted in an randomized block design with two replications at the experimental station of Huazhong Agricultural University at Wuhan (114°30′ E, 30°60′ N) in 2018 for phenotype investigation. Each line was grown in a two-row plot with 10 plants per row, with a spacing of 16.7 cm between plants and 26.6 cm between rows. The original CSSLs were grown in the same design in 2013. At the mature stage, panicle weight (PW), panicle length (PL) and primary branch (PB) were evaluated following the methods described previously^48^. Spikelet number (SN), seed setting ratio (SS), grain length (GL), grain width (GW) and thousand-grain weight (TGW) were measured using a yield traits scorer in a high-throughput phenotyping facility^49^.

### IAA and IAA-amino acid conjugate measurement

Extraction of IAA and IAA-amino acid conjugates from rice leaves and their analysis followed the method described previously^50^. Briefly, leaf samples were collected and frozen in liquid nitrogen. Approximately 100 mg powder of each sample was extracted overnight at 4 °C with 1 mL pure methanol containing 0.1 mg/L lidocaine. Following centrifugation at 10,000×*g* for 10 min, the supernatant was absorbed and filtrated using a filter (SCAA-104, 0.22-µm pore size; Shanghai ANPEL Scientific Instrument). The extracts were analyzed using liquid chromatography-mass spectrometry (ABI 4000 Q-Trap, Applied Biosystems). The measurements were repeated three times for each sample.

### Scanning electron microscopy

Young spikelets (4 days before heading) were collected for scanning electron microscopy analyses. The samples were prepared following the procedure as previously described^51^. The mastoid spacing of the lemmas of outer spikelet hulls was measured under a scanning electron microscope (JSM-6390LV, JEOL) at an accelerating voltage of 10 kV and a spot size of 30 nm. Scanning electron microscopy analysis was carried out on at least ten biological replications of mounted sample.

### Vector construction and rice transformation

A clustered regularly interspaced short palindromic repeat (CRISPR)/Cas9 vector targeting the given gene was constructed following a previously described method^15^. Briefly, a 23-nucleotide fragment (5′-ACTACAAGAGCCGCCACTTCCGG-3′) including the protospacer-adjacent motif (CGG) was designed as the target sequence using the CRISPRP tool (http://rice.hzau.edu.cn/cgi-bin/rice/CRISPR_rice). The resultant construct was introduced into *Agrobacterium tumefaciens* strain EHA105 and transferred into rice variety Zhonghua 11 (ZH11) and 9311 using the *Agrobacterium*-mediated transformation method^52^. All primers used for the transgenic tests are listed in Supplementary Table S6.

## Supplementary Information

Supplementary Information 1.

Supplementary Information 2.

## Data Availability

All data generated or analysed during this study are included in this published article and its Supplementary Information files.
