# Supplementary material for: Identification of a novel QTL and candidate gene associated with grain size using chromosome segment substitution lines in rice
Source: Sci Rep. 2021 Jan 8;11:189. doi: 10.1038/s41598-020-80667-6 (PMC7794494; doi:10.1038/s41598-020-80667-6)
Supplement: Supplementary file 1 — Supplementary Information 1. [file 41598_2020_80667_MOESM1_ESM.pdf]

Identification of a novel QTL and candidate gene associated with grain size using chromosome segment substitution lines in rice

Dianwen Wang, Wenqiang Sun, Zhiyang Yuan, Qiang Sun, Kai Fan, Chaopu Zhang, Sibin Yu*

National Key Laboratory of Crop Genetic Improvement, College of Plant Science and Technology, Huazhong Agricultural University, Wuhan 430070, China

* Correspondence: ysb@mail.hzau.edu.cn

Supplementary Figures S1-5 and Table S1-3, S6-7

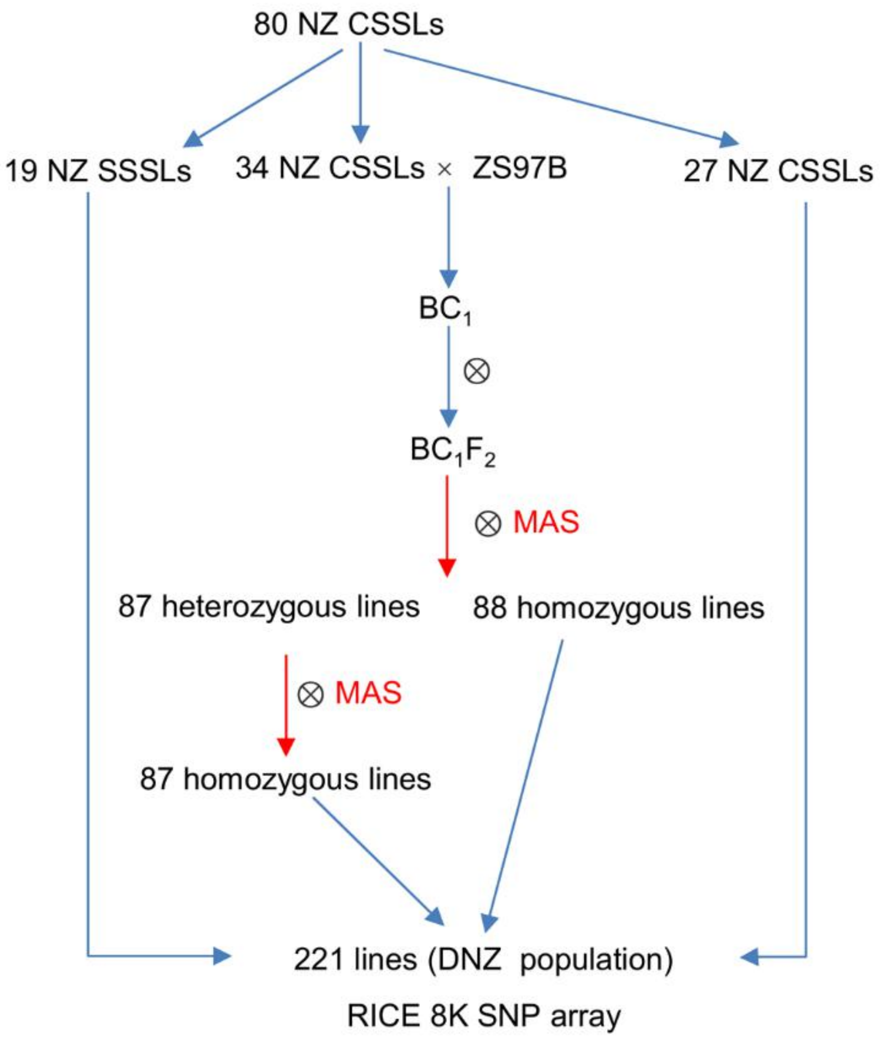

Supplementary Figure S1. Flowchart of developing chromosome segment substitution line (CSSL) population (DNZ). The red arrow indicates that the process of genotyping by marker-assisted selection approach (MAS). The black circle represents a self-cross.

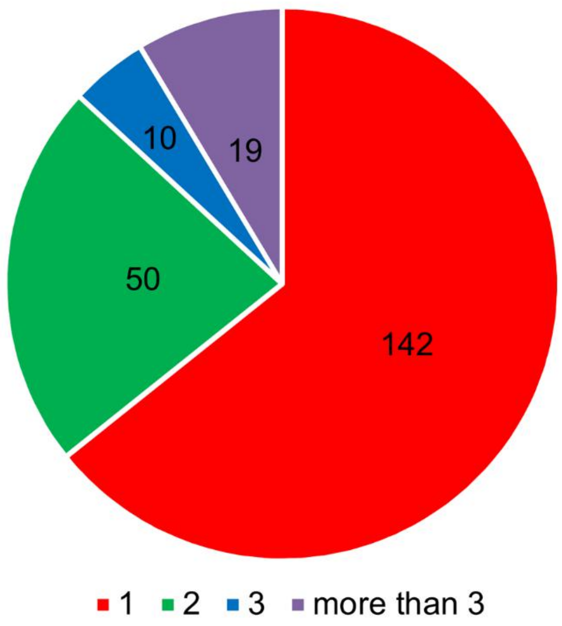

Supplementary Figure S2. Pie chart showing the number of chromosome segment substitution lines that are classed as four types in which 1, 2, 3, and more than 3 substitution segments are carried.

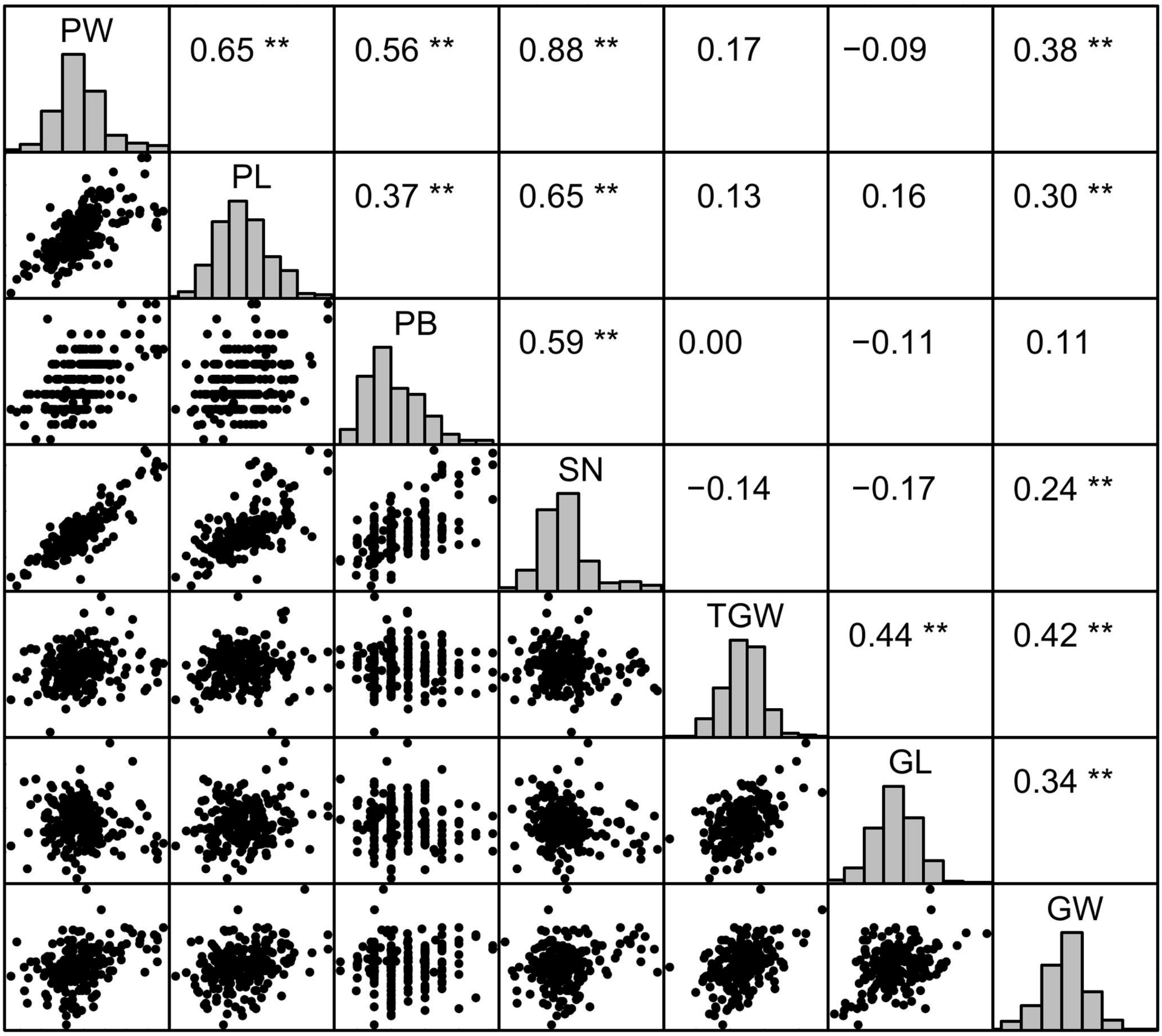

Supplementary Figure S3. Histogram of frequency distribution and correlation coefficient of seven yield-related traits in the CSSL population. GL, GW, TGW, SN, PB, PL and PW represent grain length, grain width, thousand grain weight, spikelet number, primary branch, panicle length and panicle weight, respectively. The upper panel contains correlation coefficients and the lower panel contains frequency distribution of the assayed parameters. The diagonal represents the histogram of the assayed traits. Double asterisks represent significance at $P < 0.01$.

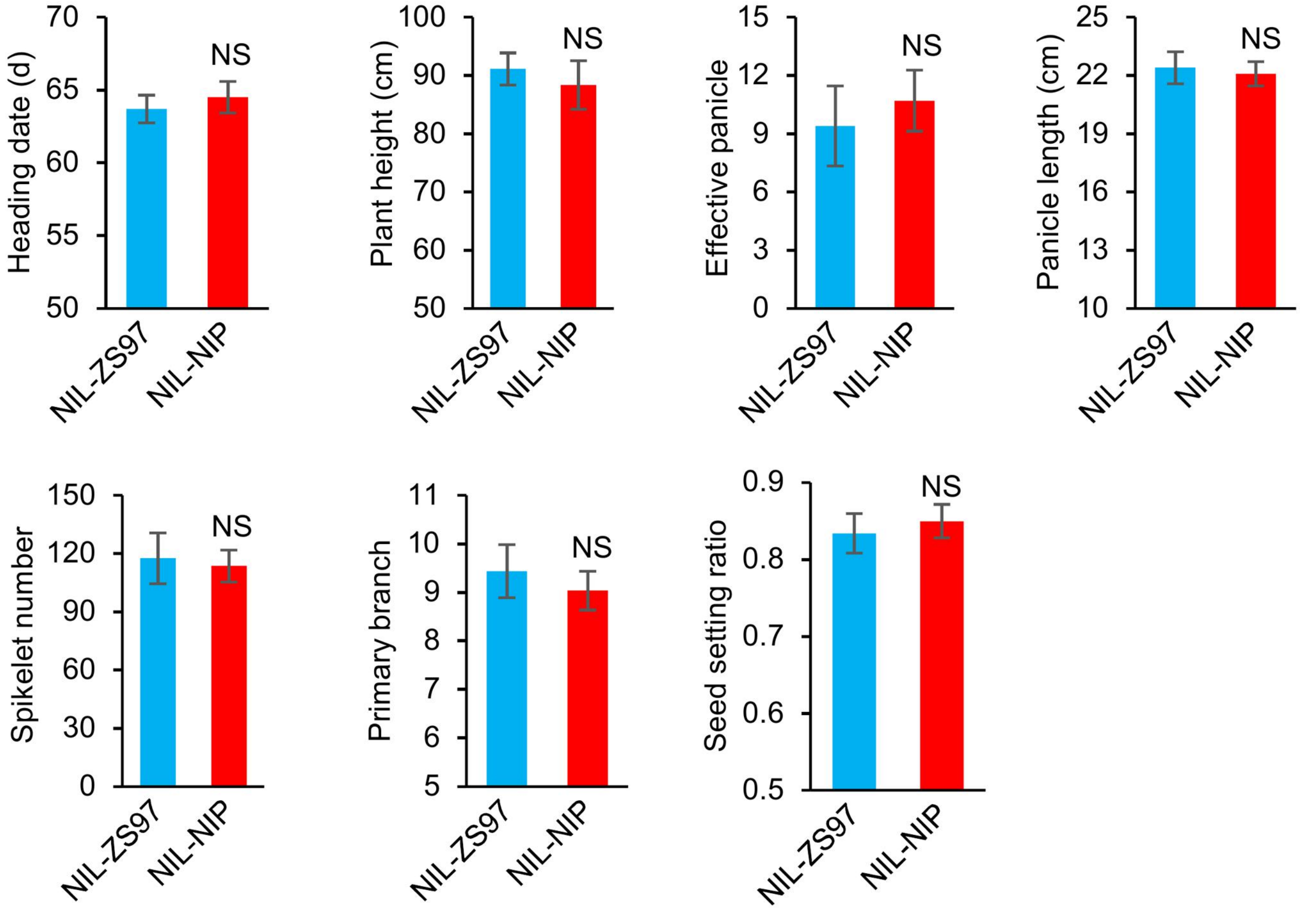

Supplementary Figure S4. Comparison of the yield-related traits in NILs of *qGL11*. Data are given as the mean and SE (n = 10). NS, no significance by Student's t-test.

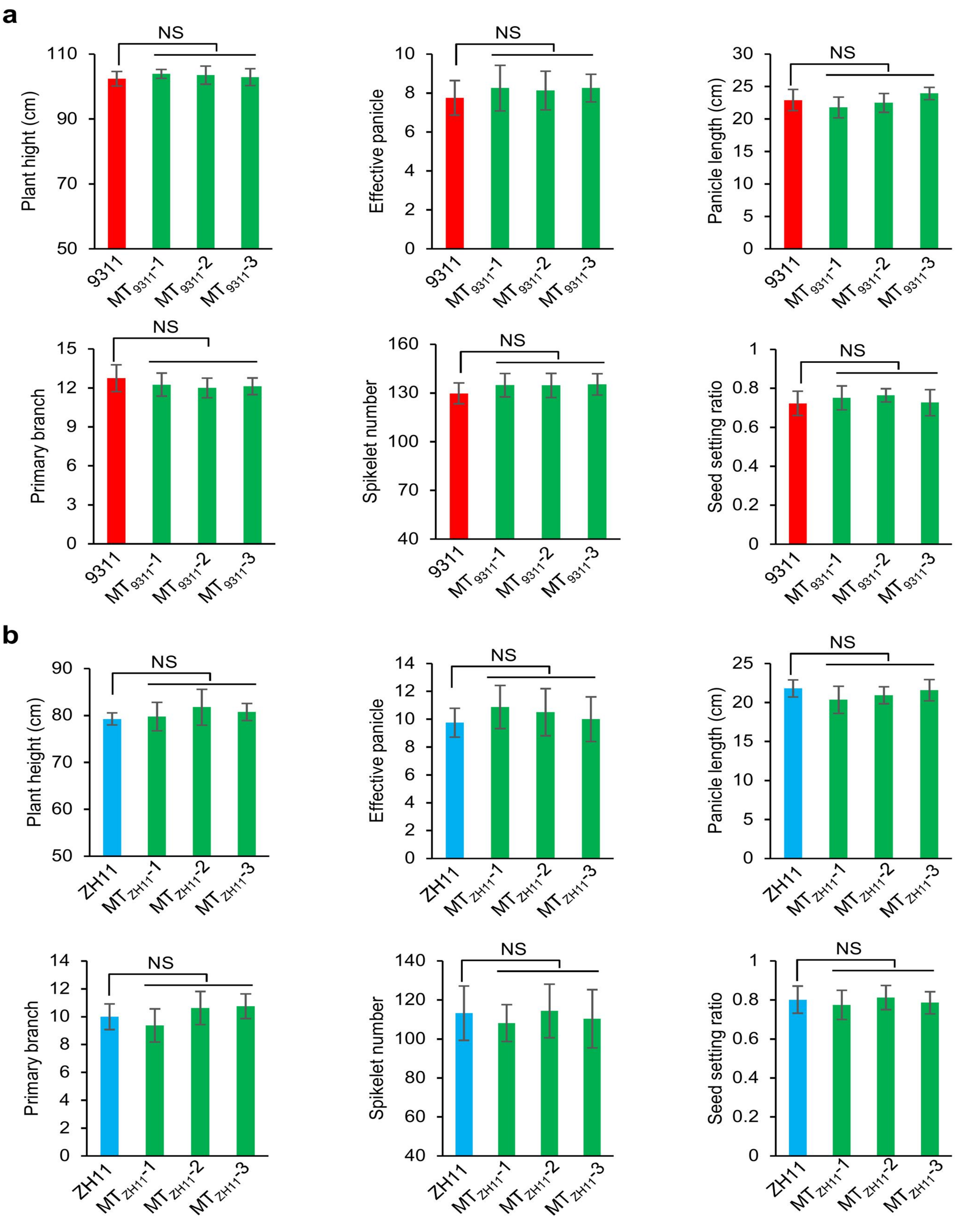

Supplementary Figure S5. Comparison of the yield-related traits in the CRISPR/Cas9 induced mutants and corresponding wild type. (a) and (b) represent the mutations of *OsGH3.13* in in 9311 and ZH11, respectively. Data are given as the mean and SE (n = 8). NS, no significant difference from the wild type by Student's t-test.

Supplementary Table S1. Distribution of polymorphic SNPs between ZS97 and NIP across the whole genome

Chr	Number of SNPs	Distance between SNPs (bp)		
		Min	Max	Median
1	451	21	2,533,264	47,336
2	415	17	1,122,836	47,535
3	401	12	931,647	53,140
4	408	15	1,660,306	33,728
5	354	15	777,527	44,729
6	381	12	1,136,278	39,963
7	367	12	1,021,740	45,705
8	260	57	1,250,806	59,528
9	160	152	3,011,420	44,429
10	137	17	1,119,873	131,061
11	158	29	1,063,284	124,376
12	118	132	1,277,886	173,441
Total	3610	12	3,011,420	50,282

Supplementary Table S2. Distribution of substitution segments and bins on the rice chromosomes in the DNZ population

Chr	Number of segments	Median of segment (Mb)	Segment coverage length (Mb)	Coverage rate of segment (%)	Number of bins	Min of bin (kb)	Max of bin (Mb)	Median of bin (kb)
1	51	1.9	39.1	90	50	22.4	8.2	406.7
2	46	1.0	35.9	100	46	12.3	4.2	408.1
3	61	2.3	35.8	98	53	25.5	5.8	478.5
4	49	3.0	34.2	97	56	1.8	6.5	290.0
5	40	2.5	29.9	100	42	29.3	3.9	481.9
6	22	2.6	24.8	81	30	2.0	6.0	399.3
7	35	1.7	29.7	100	35	44.6	4.0	457.2
8	21	3.2	28.4	100	26	69.2	7.2	620.9
9	27	5.3	23.0	100	20	34.5	6.5	532.8
10	12	2.6	23.5	100	15	166.6	3.6	1527.9
11	30	3.9	28.5	100	30	35.3	4.2	461.6
12	14	4.9	24.6	89	15	128.0	5.0	1277.9
Total	408	2.3	357.4	96	418	1.8	8.2	472.7

Supplementary Table S3. Seven panicle and grain-related traits in the DNZ population

Traits	NIP	ZS97	DNZ population		
	Mean ± SD	Mean ± SD	Mean ± SD	Range	CV
Grain length (mm)	7.8 ± 0.08	8.3 ± 0.08	8.3 ± 0.23	7.7 ~ 9.2	2.8
Grain width (mm)	3.2 ± 0.05	3.5 ± 0.05	3.4 ± 0.11	3.1 ~ 3.8	3.2
Thousand-grain weight (g)	21.3 ± 0.56	25.4 ± 0.89	24.8 ± 1.19	20.5 ~ 29.4	4.8
Panicle length (cm)	17.9 ± 0.52	22.8 ± 0.78	22.8 ± 1.54	18.8 ~ 27.8	6.7
Primary branch	10 ± 0.88	10 ± 0.88	10.6 ± 1.71	7 ~ 16	16.1
Spikelet number	87 ± 6.2	120 ± 7.7	127.2 ± 24.7	69 ~ 216	19.4
Panicle weight (g)	1.5 ± 0.17	3 ± 0.29	2.9 ± 0.63	1.3 ~ 4.9	21.5

Phenotype data of NIP and ZS97 are given as mean with SD (n = 10)， SD and CV stand for standard deviation and coefficient of variation, respectively.

Supplementary Table S6. Primers used in the study

Primer	Forward primer sequence (5'-3')	Purpose
M5F	AGCATGATGTTTCATACGTGA	mapping *qGL11*
M5R	ATGGGATATTGCTTCACCAC	
M9F	AAGGTTGACAAGGACAGAAG	
M9R	TCGCAGGAATGGATAAAA	
M8F	ACAGTATTCCGTAGGCACGG	
M8R	GCTCCATGAGGGTGGTAGAG	
M10F	GTCAACGGTGTCAAACATTTCG	
M10R	TTAGGAGTGGCCTGCTATGTATGC	
M7F	CGACACAGTTACAAAGCCACAGC	
M7R	TGGGAGGACGTTGATATGTCTCG	
M11F	GATGATCAGGAGGCAACAC	
M11R	CGTGATCACCTTACAATCCA	
M12F	CCCTCCATTTCAGATTATACG	
M12R	TTGATACTTCATCCGTTTAAGG	
M3F	TAGCTACATTGCCGTGAGAG	
M3R	TGGTTATTTGCGATAGGACA	
ID5L	GGGTCAAAGGTCAGTGATGT	
ID5R	TGTACCTTGGCAGTTATGGGT	
ID1F	TCACTGTGCAGAATCGACCA	
ID1R	GGGATGTCTCTTGCAGCATG	
ID3F	TTTCTTCAGGTCGCAGTG	
ID3R	CAACAGGTCCAATTTTCG	
ID2F	CCCAGTGGTTATAGATGG	
ID2R	TTTAGCAAGTAGTGTCCC	
M1F	CACTTCAACTTCCTCCTCCTTTCC	
M1R	AGTGTCGGTCTTGTGGTTCTTGC	
M2F	AAGAGGAGGAGGAGAGAGAAGC	
M2R	CAGAGAGTTGTACACATTCTGAGC	
M4F	GCGGACACACCAGAGAATAAGC	
M4R	GTGCTGTCCTGTCCTTGAATCC	
M6F	TTCCCTCCTATGTAGCATTAGC	
M6R	ATGGTGGAGAGTACTTCATTCC	
ID6F	TCGATAGAAATCAACCCT	
ID6R	AGATATCATTAGCGGAGA	
ID7F	CAAGCGTCACTACTACAGAT	
ID7R	GCATTCGCCTAGTTCCAG	
ID8F	AGCCCAAGAACAAGAGGA	
ID8R	AGGTCCTGCCGAGATTTA	
ID9F	ATCGGTGGATGGATGGCT	
ID9R	GGGACCAGGAGGCTTCAC	
ID10F	TTGCTTGGTGAGATTGAC	
ID10R	GACATCCTAAACCCTCCC	
Os11g0528200RTF	TGATTTCTGGCTATTATGCG	Realtime PCR
Os11g0528200RTR	GGACTACAAACCAAAGGACC	
Os11g0528300RTF	GCATTGAAGGACCAAAGTA	
Os11g0528300RTR	CTATAGGGGCAACAAAACA	
Os11g0528400RTF	TACACTATCCGTGCTTGGT	
Os11g0528400RTR	CATCTGGTGTCAGGCTTT	
Os11g0528500RTF	GTTTCTTGTGCTGCGGTGAG	

Os11g0528500RTR	GCGTGCAGAACGATGACG	
Os11g0528700RTF	CTTCTCCACCTTGATGAACTCG	
Os11g0528700RTR	CTCTACAACCTCCTCGTCCC	
UBQ-F	AACCAGCTGAGGCCCAAGA	
UBQ-R	ACGATTGATTTAACCAGTCCATGA	
U3F	CCCCTTTCGCCAGGGGTACCGTAATTCATCCAGGTCTCCAAG	Vector construction
U3R	TACGAATTCGAGCTCGGTACCGCTGTGCCGTACGACGGTACG	
OsGH3.13-U3R	ACTACAAGAGCCGCCACTTCGTTTTAGAGCTAGAAATAGCAAGTTA	
OsGH3.13-U3F	GAAGTGGCGGCTCTTGTAGTGCCACGGATCATCTGCACAACTC	
CAS9F	GAGCGGATAACAATTTCACACAG	Transgenic detection
CAS9R	TCTATGTTACTAGATCGGGAATTCA	
SCROsGH3.13	GGGGCTCGTGTACCGCGTGT	
SCROsGH3.13	CCGTCGTGATCTCCGGCTTCA	

Supplementary Table S7. Sequence comparison of the 3-kb promoter region and coding region of *OsGH3.13*^{NIP} and *OsGH3.13*^{ZS97}

Position	Sequence of NIP (5'-3')	Sequence of ZS97 (5'-3')	Conserved motifs
-38	TGAGAACTAGG	TGAGAGCTAGG	
-61	AAGAA(CA)$_8$CCAAA	AAGAA(CA)$_7$CCAAA	
-127	TAGAGACCAAG	TAGAG**GCC**AAG	GCC (Ethylene-response element)
-179	TTGTCCGACTA	TTGTCTGACTA	
-185	CTCTCTTTGTC	CT**CTCC**TTGTC	CTCC-motif
-387	TCCTC - AAAAA	TCCTCTAAAAA	
-727	AACATAAAGAG	AACATCAAGAG	
-898	AATACTGGAGT	AATACAGGAGT	
-1327	AACAG(A)$_9$TACTG	AACAG(A)$_{10}$TACTG	
-1671	AGCTGCAGCAG	AGCTGAAGCAG	
-1892	TTAC**TTCTT**TC	TTACTCCTTTC	AAGAA-motif
-1985	CTATGACATAT	CTATGTCATAT	
-2037	CATATTGTGTG	CATATGGTGTG	
-2458	TGCAGAATACA	TGCAG**GATACA**	**GATACA (auxin response element)**
-2709	TTATAGCATTT	TTATAACATTT	
-2760	TCCCAACACTG	TCCCATCACTG	
-2821	GAACCGAATCT	GAAC**CAAAT**CT	CAAT-box
29	CCC	CTC	Non-synonymous variation
260	GTC	GCC	Non-synonymous variation
521	TCA	TCG	Synonymous variation
745	GGG	GGA	Synonymous variation
769	ACG	ACT	Synonymous variation
943	TCC	TCT	Synonymous variation
5424	GAC	AAC	Non-synonymous variation

The position of the start codon (ATG) of *OsGH3.13* is recorded as 1. Natural variations in the 3-kb promoter region and coding region are shown in red font; subscript digit indicates the number of base repeats in parentheses. Conserved motifs are predicted based on the PlantCARE database, which contains natural variations are underlined.
